# Immunomodulation by fish-oil containing lipid emulsions in murine acute respiratory distress syndrome

**DOI:** 10.1186/cc13850

**Published:** 2014-04-29

**Authors:** Matthias Hecker, Juliane Ott, Christoph Sondermann, Martina Barbara Schaefer, Martin Obert, Andreas Hecker, Rory E Morty, Istvan Vadasz, Susanne Herold, Bernhard Rosengarten, Martin Witzenrath, Werner Seeger, Konstantin Mayer

**Affiliations:** 1 University of Giessen + Marburg Lung Center (UGMLC), University Hospital of Giessen, Justus-Liebig-University of Giessen, Klinikstr. 33, D - 35392 Giessen, Germany; 2 Department of Neuroradiology, University Hospital of Giessen, Justus-Liebig-University of Giessen, Giessen, Germany; 3 Department of General and Thoracic Surgery, University Hospital of Giessen, Justus-Liebig-University of Giessen, Giessen, Germany; 4 Department of Lung Development and Remodelling, Max Planck Institute for Heart and Lung Research, Bad Nauheim, Germany; 5 Department of Neurology, University Hospital of Giessen, Justus-Liebig-University of Giessen, Giessen, Germany; 6 Charité - Universitätsmedizin Berlin, Medizinische Klinik mit Schwerpunkt Infektiologie und Pneumologie, Berlin, Germany

## Abstract

**Introduction:**

Acute respiratory distress syndrome (ARDS) is a major cause of mortality in intensive care units. Patients with ARDS often require parenteral nutrition with lipid emulsions as essential components. Besides being an energy supply, these lipid emulsions might display differential modulatory effects on lung integrity and inflammation.

**Methods:**

In a pre-emptive strategy, we investigated the impact of three different intravenously infused lipid emulsions on lung morphology, leukocyte invasion, protein leakage and cytokines in a murine model of ARDS. Mice received an infusion of normal saline solution, a pure long-chain triglycerides (LCT) emulsion, a medium-chain triglycerides (MCT) containing mixed emulsion (LCT/MCT), or a fish oil (FO) containing mixed emulsion (LCT/MCT/FO) before lipopolysaccharide (LPS) challenge.

**Results:**

Mice pre-infused with fish oil-containing lipid emulsion showed decreased leukocyte invasion, protein leakage, myeloperoxidase activity, and cytokine production in their alveolar space after LPS challenge compared to mice receiving LCT or LCT/MCT. In line with these findings, lung morphology assessed by histological staining after LPS-induced lung injury improved faster in the LCT/MCT/FO group. Concerning the above mentioned parameters, no significant difference was observed between mice infused with LCT or the combination of LCT and MCT.

**Conclusion:**

Fish oil-containing lipid emulsions might exert anti-inflammatory and pro-resolving effects in the murine model of acute lung injury. Partial replacement of n-6 fatty acids with n-3 fatty acids may thus be of benefit for critically ill patients at risk for ARDS which require parenteral nutrition.

## Introduction

Acute respiratory distress syndrome (ARDS) is a common clinical disorder characterized by alveolar epithelial and endothelial injuries leading to the development of protein-rich non-cardiogenic pulmonary edema, elevation of pulmonary artery pressure and, finally, respiratory failure [1]. The incidence of ARDS was 4.5 to 7.1% in all patients admitted to an intensive care unit (ICU) [2,3]. This percentage increases to 12.5% when considering only patients treated longer than 24 h in the ICU [3]. Despite a multitude of promising pharmacological approaches being successful in animal studies, there is at present no proven pharmaceutical option available for ARDS patients. This is reflected by the still unacceptable high mortality rate of 30 to 40% [4]. The pathophysiology of ARDS is complex and still not fully understood. The acute phase of ARDS is characterized by a widespread disruption of the alveolar-capillary barrier leading to increased vascular permeability, neutrophil invasion into the interstitial and alveolar space, and the formation of pro-inflammatory mediators such as cytokines and eicosanoids [1,4].

Lipid emulsions are considered as essential components of clinical parenteral nutrition regimens applied to critically ill patients. Besides providing adequate caloric support, several studies indicate the immunomodulatory properties of lipid emulsions, as shown by their ability to alter cytokine release, to modify leukocyte function, and to influence the generation of lipid mediators that display both pro- and anti-inflammatory properties [5-7]. For a long time supplementation of fatty acids was exclusively relying on soybean or safflower oil-based long-chain triglycerides (LCT), which contain a large amount of linoleic acid (LOA, 18:2), a n-6 polyunsaturated fatty acid (PUFA), serving as a precursor of arachidonic acid (AA). Rapid and extended infusion of such lipid emulsions may thus increase the plasma concentration of free arachidonic acid by one order of magnitude, leading to a modulation of the eicosanoid profile, deterioration of the oxygenation index and alteration of the ventilation-perfusion matching of the lung. The combination of LCT with medium-chain triglycerides (MCT) in a 1:1 ratio was introduced into nutrition regimes as MCT were cleared rapidly from the serum, and displayed less adverse liver outcomes and reduced the provision of n-6 PUFA by 50% [8-10]. The administration of n-3 PUFA, as fish oil (FO), offers a novel promising strategy to enrich nutrition regimes as it has been shown to modulate excessive inflammatory reactions in animals, healthy volunteers and subjects in clinical trials [11]. Nevertheless, the clinical use of n-3 PUFA in ARDS patients is at present debated. In critically ill patients suffering from sepsis or ARDS, enteral supplementation of n-3-based lipid emulsions reduced mortality and displayed anti-inflammatory properties [5]. On the other hand, a large multi-center study, conducted by the ARDSnet, was recently published and investigatedg the effects of an enteral supplementation of n-3 fatty acids in ARDS patients [12]. The study was stopped early because of futility; it displayed a higher rate of complications in the group receiving n-3 fatty acids. Due to the inconsistency of data concerning the enteral use of n-3 fatty acids in ARDS, there is an on-going debate in the scientific community with no final recommendation available at the moment.

Despite our better understanding of the pathophysiological effects caused by the currently available lipid emulsions in clinical use for parenteral nutrition, especially the value of novel emulsions, such as mixed lipid emulsions containing MCT/LCT and/or FO, their use in ARDS requires further investigation. Due to the novelty of these lipid emulsions in clinical use there is still a paucity of data in experimental models and clinical settings.

In the present study, we investigate the effects of conventional LCT-based lipid emulsions in the murine model of endotoxin-induced ARDS compared to mixtures of MCT/LCT with or without FO supplementation. For this purpose, we make use of a continuous long-term lipid infusion system, followed by endotoxin challenge and subsequent *in vivo* and *in vitro* analyses. For our study, we chose a “pre-emptive” strategy by administration of lipid emulsions prior to induction of lung injury. The background of this concept is the choice of an appropriate lipid emulsion for patients in need of parenteral nutrition and with an expected trauma/operation or at risk, for example, of aspiration-induced respiratory distress.

## Materials and methods

### Reagents

A soybean-based lipid emulsion (LCT; Lipoven 20%®) was purchased from Fresenius-Kabi (Bad Homburg, Germany). Both the mixtures of LCT (50%) and MCT (50%) (LCT/MCT; Lipofundin 20%®) and LCT (40%)/MCT(50%)/FO(10%) (Lipoplus 20%®) were obtained from B.Braun (Melsungen, Germany). Analysis of the fatty acid composition of the lipid emulsions is given in Table 1. Chemicals of the highest purity were obtained from Merck (Darmstadt, Germany). Lipopolysaccharides (LPS, O111:B4) from *E. coli* were obtained from Sigma-Aldrich (Dreisenhofen, Germany).

**Table 1:** Fatty acid composition (g/L) of the lipid emulsions used assessed by gas chromatography

**Fatty acid**	**LCT**	**LCT/MCT**	**LCT/MCT/FO**
**8:0**	0.00	57.21	55.42
**10:0**	0.00	41.78	41.45
**16:0**	21.56	10.27	10.57
**16:1**	0.00	0.00	0.21
**18:0**	11.20	7.67	7.43
**18:1**	49.52	26.85	24.09
**18:2**	106.07	48.57	41.20
**18:3**	11.47	6.05	4.49
**20:4**	0.18	0.40	0.76
**20:5**	0.00	0.00	5.12
**22:5**	0.00	0.00	1.60
**22:6**	0.00	0.19	4.54

### Animals and experimental protocol

All animal experiments were performed in Giessen; animal experiments were approved by the animal protection branch of the Regierungspräsident Gießen and the animal protection representative of the University of Giessen. BALB/c mice were kept under standard conditions with a 12 hour day: night cycle under specific pathogen-free conditions. Animals 13 to 15 weeks old (22 to 24 g in weight) were used for the experiments. The implantation of a jugular vein catheter and subsequent adaptation to an osmotic mini-pump (Alzet, Cupertino, CA, USA) was performed as described previously [13].

Seven days after central venous catheter implantation in mice, an exchange of pumps was performed. Then, either 200 μl per day of LCT, LCT/MCT, LCT/MCT/FO or normal saline (NaCl) were infused over three days with the mice being allowed access to water and chow *ad libitum*. The amount of lipids infused is equivalent to 1.5 g/kg/d. However, the energy expenditure of mice is nearly three times higher than that in humans. Therefore, the infused lipids were considered to be close to the lower limits of the recommended amount of lipids in parenteral nutrition. While receiving infusions, mice were subjected to low dose unfractionated heparin injected subcutaneously.

### LPS-induced acute lung injury in a murine model

After completion of the infusion regimen, mice were anesthetized with xylazine/ketamine, a small catheter was inserted in the trachea, and LPS (0 or 10 μg in 200 μl normal saline/mouse) was instilled by using a Microsprayer (Penn-Century Inc., Philadelphia, PA, USA). A total of 4, 24 or 48 hours after LPS-application, mice were anesthetized as described before and volumetric computed tomography of the lung was performed. After that, mice were sacrificed with an overdose of anesthesia (xylazine/ketamine), and their lungs were either taken for further examination or a bronchoalveolar lavage (BAL) was performed as described previously [14].

### Assessment of lung edema

Lung edema was estimated by protein determination in BAV according to Lowry [15].

### Histological assessment of lung morphology

For histochemical assessment of lung morphology, tissue was fixed, embedded and stained with hematoxylin and eosin as previously described [16].

### Volumetric computer tomography

Mice were anesthetized and examined by high-resolution flat-panel volumetric computed tomography (fpvCT). This CT is exclusively used for experimental trials in animals. Examinations are acquired at 120 kV and 40 mA. One thousand projection images are taken within one gantry rotation of eight seconds duration. A matrix of 1,024 columns × 360 rows is the read-out of the flat-panel detector. Images are reconstructed using a cone-beam filtered back-projection algorithm with an isotropic voxel size of 0.05 mm.

### BAL leukocytes counts

Mice were killed by an overdose of anesthesia, and BAL was performed *in situ* as described [14]. Alveolar-recruited leukocytes recovered from the lungs of LPS-challenged and control mice were counted in a counting chamber under a light microscope. Differential leukocyte counting analysis was performed after H&E staining.

### Enzyme-linked immunosorbent assay (ELISA)

Tumor-necrosis factor (TNF)-α, macrophage inflammatory protein (MIP)-2, prostaglandin (PG)E_2_ (all purchased from R&D Systems, Wiesbaden, Germany), and thromboxane (Tx)B_2_ (Assay Designs, Ann Arbor, MI, USA) from BAL were determined by ELISA, according to the manufacturers’ instructions.

### Myeloperoxidase assay

Lung myeloperoxidase (MPO) was determined as an index of tissue neutrophil accumulation after LPS challenge as previously described [13]. After weighing the lungs stored at -80°C, the frozen lungs were homogenized, sonicated and centrifuged at 25,000 × g. MPO activity was calculated from the change in absorbance (460 nm) resulting from decomposition of H_2_O_2_ in the presence of o-dianisidine.

### Determination of free fatty acids in plasma

Plasma was collected directly after sacrifice by venous puncture and free fatty acids were determined by gas chromatography as described [17].

### Statistics

Data are given as the mean ± SEM. Independent experiments (n = 6 to 8) were performed per group and time-point. Two-way analysis of variance (ANOVA) was performed to test for differences between different infusion groups. *Post-hoc* analysis was carried out using Student-Newman-Keuls test. Probability (*P*) values <0.05 were considered to indicate statistical significance. Analysis was carried out using SigmaStat® 3.5 for Windows (Systat Software GmbH; Erkrath, Germany).

## Results

### Effects of lipid emulsions on lung morphology and inflammation after induction of ARDS

**Figure 1:**
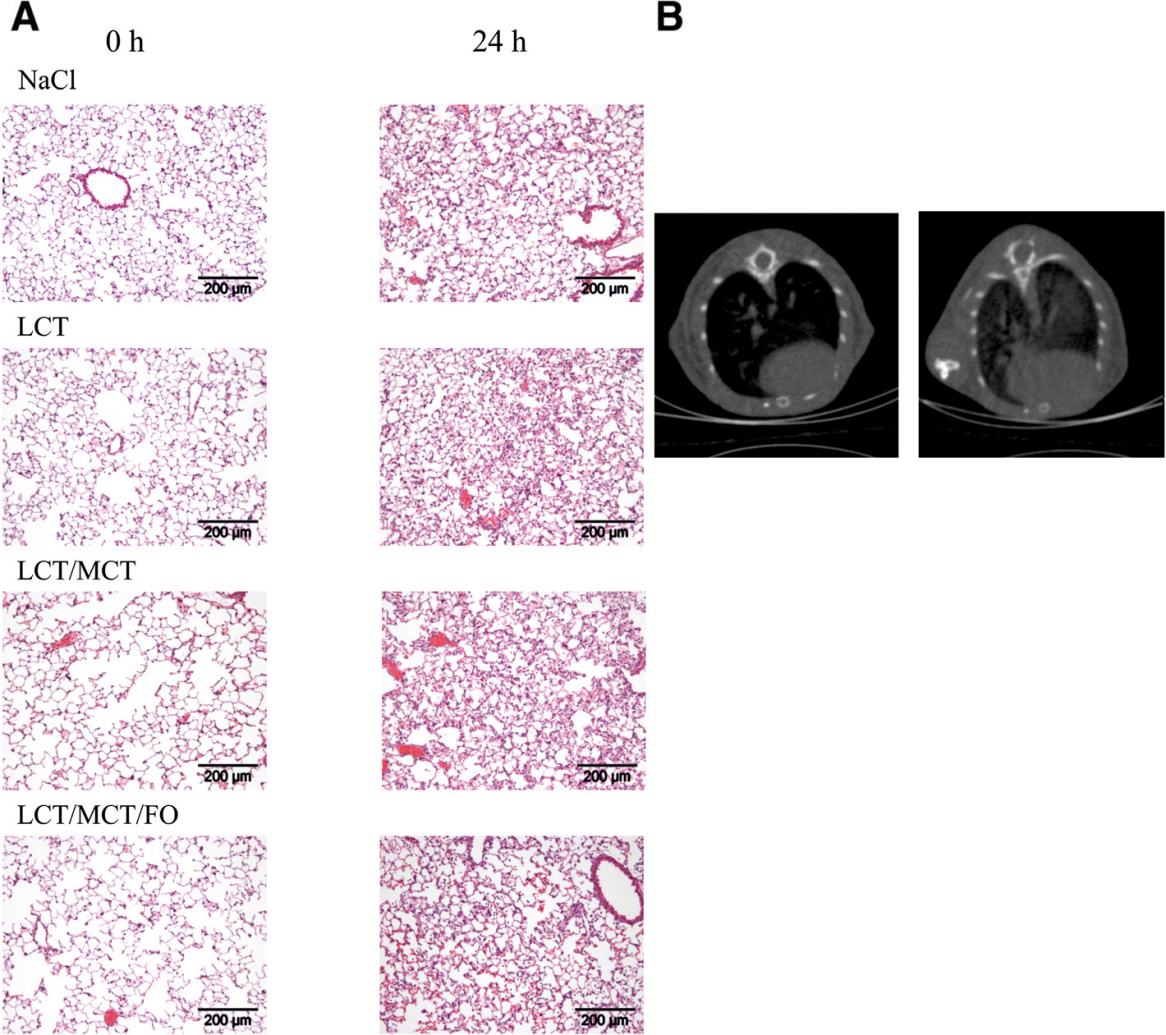
**Morphological effects of lipid emulsions after induction of ARDS. A**. Histological assessment of lung morphology before (0 h) and 24 h after LPS-induced ARDS. **B**. Representative example of high-resolution flat-panel volumetric computed tomography of lungs before (left) and 24 h after induction of ARDS (right). ARDS, acute respiratory distress syndrome; LPS, lipopolysaccharide.

Mice were infused with NaCl, LCT, LCT/MCT or LCT/MCT/FO over three days, followed by intra-tracheal application of LPS 24 h prior to being sacrificed. Lung morphology was assessed by histology to evaluate the inflammatory effect. Before challenge, lungs of mice from all treatment groups displayed a similar histological pattern (Figure 1A). After LPS instillation, lungs of animals infused with NaCl showed a marked increase in leukocyte invasion into the alveolar space and interstitial edema as signs of pulmonary inflammation and damage. These morphological features of acute lung injury were even more pronounced in the LCT and LCT/MCT group. In contrast, leukocyte invasion and edema formation were ameliorated in mice receiving LCT/MCT/FO.In a second approach, lung morphology and the extent of lung injury was assessed by high-resolution flat-panel volumetric computed tomography (Figure 1B). Independent of the lipid infusions applied, none of the lungs showed pathologies such as relevant pleural effusions, pneumothorax, or significant atelectasis before and after injury.

### Effect of lipid emulsions on alveolar recruitment of leukocytes in LPS-induced acute respiratory distress syndrome

**Figure 2:**
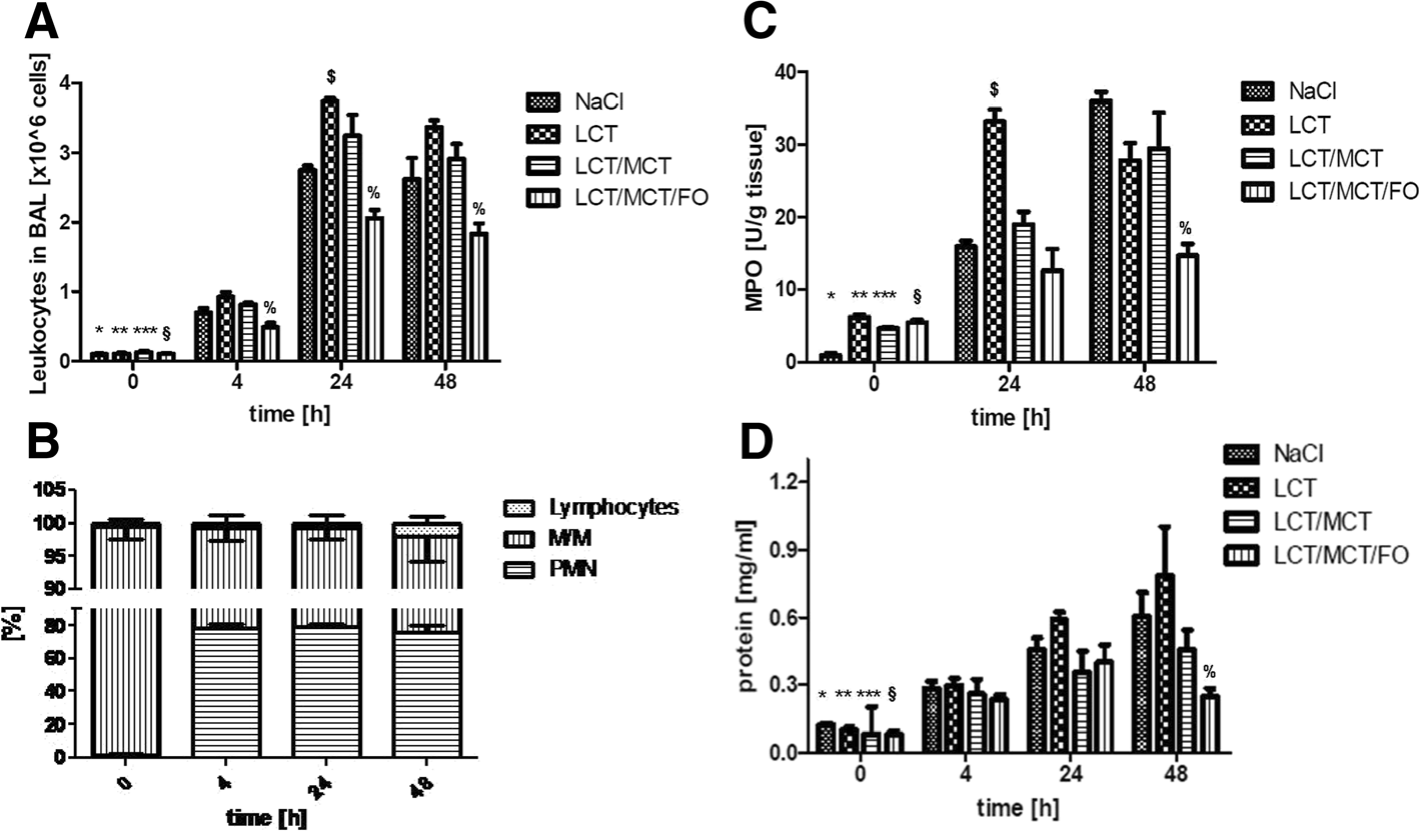
**Invasion of leukocytes into the alveolar space in LPS-induced ARDS. A**. In ARDS, leukocytes migrated into the alveolar space in all groups (*, NaCl; **, LCT; ***, LCT/MCT; § LCT/MCT/FO). After 24 h of LPS-stimulation the LCT group displayed an increased number of leukocytes compared to the NaCl group ($). Mice receiving LCT/MCT/FO infusions displayed the lowest number of alveolar leukocytes at all time-points after LPS stimulation compared to all other groups (%). **B**. The percentage of lymphocytes, monocytes/macrophages (M/M) and polymononuclear cells (PMN) was calculated in the BAL of all treatment groups and time-points. The time course for the NaCl group is shown but the same pattern was detectable for all other groups. **C**. Myeloperoxidase (MPO) activity as a marker of PMN invasion increased significantly after 24 h and 48 h in the NaCl group (*) and all other groups compared to control (**, LCT; ***, LCT/MCT; § LCT/MCT/FO). A total of 24 h after ARDS, the LCT group displayed the highest MPO activity of all groups ($). After 48 h, MPO activity was the lowest in animals receiving LCT/MCT/FO as compared to all other groups (%). **D**. In all groups a significant increase in protein extravasation 4 h, 24 h and 48 h after induction of ARDS could be observed, which was valid for NaCl (*) and the other groups (**, LCT; ***, LCT/MCT; § LCT/MCT/FO). After 48 h of LPS challenge, the mice receiving LCT/MCT/FO showed significantly reduced levels of protein leakage as compared to NaCl-, LCT-, and LCT/MCT-infused mice at this time-point (%). Data are given as mean ± SEM (n = 6 to 8 independent experiments each). All markers indicate *P* <0.05. ARDS, acute respiratory distress syndrome; BAL, bronchoalveolar lavage; FO, fish oil; LCT, pure long-chain triglycerides; LPS, lipopolysaccharide; MCT, medium-chain triglycerides; MPO, myeloperoxidase.

Mice were infused for three days with normal saline solution (NaCl control), LCT, LCT/MCT or LCT/MCT/FO, respectively, followed by intra-tracheal application of 10 μg LPS, and the performance of a BAL 4 hours, 24 hours or 48 hours later. Without LPS-challenge, we found 0.10 ± 0.03 × 10^6^ leukocytes in BAL fluid without significant variation between the NaCl and lipid infusion groups (Figure 2A). After stimulation with LPS, leukocytes migrated into the alveolar space with their numbers in BAL fluid rising to 0.70 ± 0.16 × 10^6^ cells (*, *P* <0.05 vs. control) after 4 h, to 2.75 ± 0.25 × 10^6^ 24 h after LPS instillation (*, *P* <0.05 vs. control), and to 2.62 ± 0.71 × 10^6^ after 48 h of stimulation (*, *P* <0.05 vs. control). The same significant effects were observed for the different lipid emulsions 4 h, 24 h and 48 h after induction of ARDS compared to control (**, LCT; ***, LCT/MCT; § LCT/MCT/FO). For all lipids and NaCl the leukocyte counts were significantly different between the time-points except comparing 24 h and 48 h. After 24 h of LPS-stimulation the LCT group displayed an increased amount of alveolar leukocytes compared to the NaCl group ($, *P* <0.05). For the other time-points, both LCT- and LCT/MCT-infused animals showed no significant difference on LPS-induced leukocyte invasion as compared to the NaCl group. Mice receiving LCT/MCT/FO infusions displayed the lowest number of alveolar leukocytes at all time-points after LPS stimulation in comparison to control animals and mice receiving the other lipid emulsions (%, *P* <0.05). Next, we performed a differential cell count of BAL leukocytes to elucidate the percentage of lymphocytes, monocytes/macrophages (M/M) and neutrophils (PMN). In the NaCl group, LPS instillation induced a dramatic decline in M/M, whereas relative PMN amount strongly increased and lymphocytes remained unchanged. Interestingly, this cellular pattern was valid for all different time-points. The differential leukocyte count in the BAL of mice treated with the different lipid emulsions showed no significant difference compared to NaCl (Figure 2B).

### Accumulation of neutrophils in lung tissue

MPO activity was measured before and 24 h and 48 h after LPS-challenge in all groups to assess neutrophil accumulation in lung tissue. In lungs of mice without LPS exposure, MPO activity did not differ significantly among the different treatment groups (Figure 2C). After instillation of 10 μg LPS, MPO activity increased significantly to 15.9 ± 0.7 units/g after 24 h and to 37.0 ± 1.4 units/g after 48 h in the NaCl group (*, *P* <0.05). The same significant effects were observed for the different lipid emulsions 24 h and 48 h after induction of ARDS compared to control (**, LCT; ***, LCT/MCT; § LCT/MCT/FO). A total of 24 h after ARDS, the LCT group displayed the highest MPO activity of all groups ($, *P* <0.05). After 48 h, MPO activity was the lowest in animals receiving LCT/MCT/FO as compared to NaCl and the other treatment groups (%, *P* <0.05).

### LPS-induced protein extravasation in ARDS

Baseline protein concentrations in the BAL fluids showed no significant differences among the different treatment regimes (Figure 2D). Compared to these values we could observe a significant increase in protein extravasation in all respective groups 4 h, 24 h and 48 h after induction of ARDS, which was valid for NaCl (*, *P* <0.05) and the different lipid emulsions (**, LCT; ***, LCT/MCT; § LCT/MCT/FO). A total of 48 h after LPS challenge, mice receiving LCT/MCT/FO showed significantly reduced levels of protein leakage as compared to NaCl-, LCT- and LCT/MCT-infused mice at this time-point (%, *P* <0.05).

### Cytokine and lipid mediator generation in ARDS

**Figure 3:**
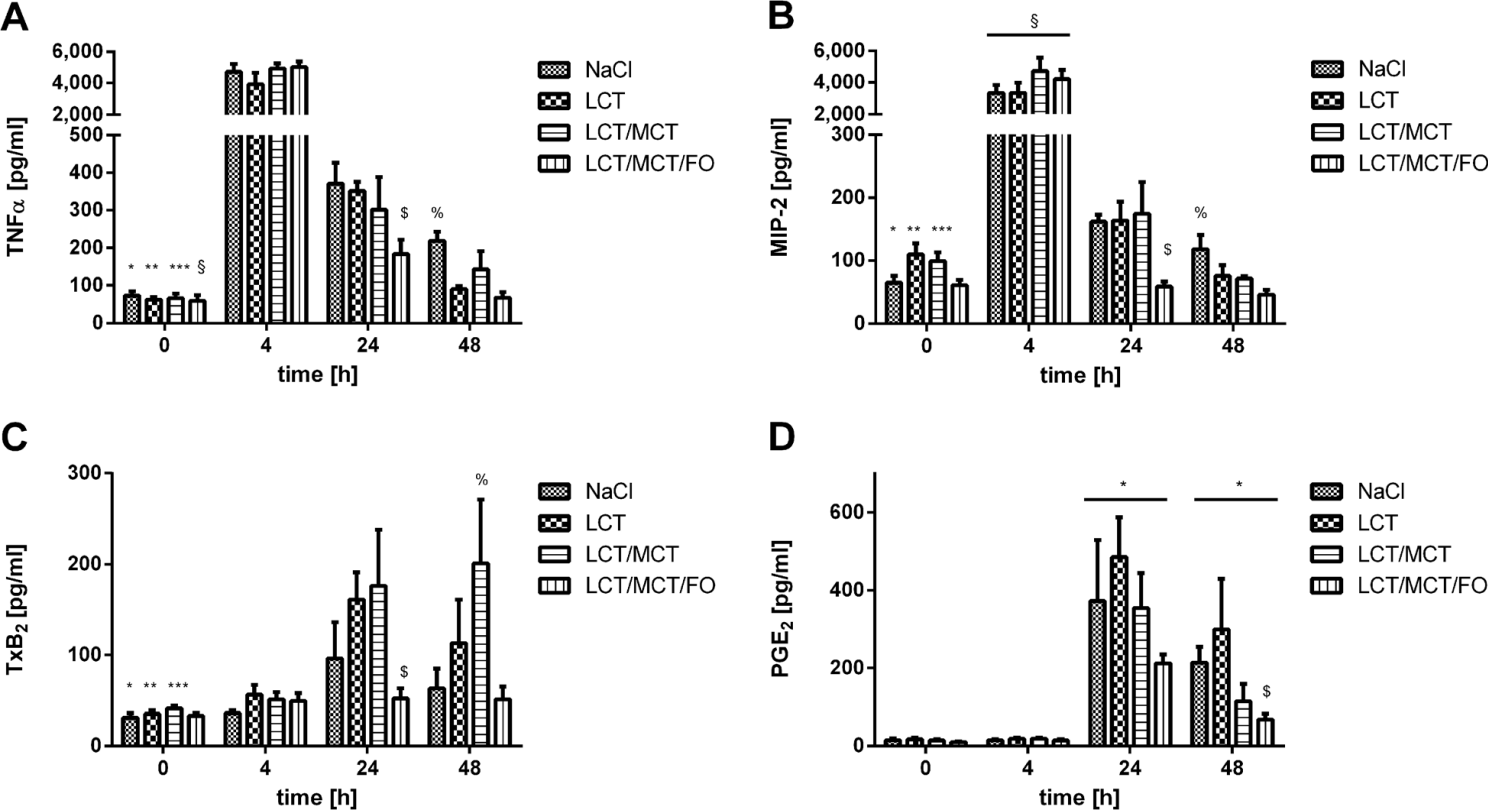
**Effect of lipid emulsions on mediators in the BAL after LPS-induced ARDS. A**. For all treatment groups (*, NaCl; **, LCT; ***, LCT/MCT; §, LCT/MCT/FO), TNF-α concentrations were increased 4 h after ARDS induction. After 24 h of injury, the LCT/MCT/FO group showed reduced levels as compared to NaCl and LCT ($). The highest concentrations were found in the NaCl group 48 h after ARDS induction (%). **B**. Highest MIP-2 concentrations in the NaCl group were measured 4 h after LPS application (*). Under basal conditions, LCT (**) and LCT/MCT (***) displayed elevated levels compared to NaCl. The highest values were measured after 4 h in all groups (§). Animals infused with LCT/MCT/FO displayed the lowest MIP-2 levels at 24 h compared to the other groups ($). After 48 h, the highest concentrations were detectable for NaCl in comparison to LCT and LCT/MCT/FO (%). **C**. A significant increase of TxB2 in the NaCl group was observed after 24 h compared to baseline (*). After 24 h and 48 h, TxB2 was rising in the LCT- (**) and LCT/MCT-group (**) compared to baseline. After 24 h, reduced levels were detected in the LCT/MCT/FO group as compared to LCT and LCT/MCT ($). After 48 h the highest concentrations were measured in animals receiving LCT/MCT compared to NaCl and LCT/MCT/FO (%). **D**. PGE2 values were elevated in all treatment groups compared to 0 h and 4 h (*). Mice treated with LCT/MCT/FO showed significantly reduced PGE2 concentration after 48 h as compared to NaCl and LCT ($). Data are given as mean ± SEM (n = 6 to 8 independent experiments each). All markers are *P* <0.05. ARDS, acute respiratory distress syndrome; FO, fish oil; LCT, pure long-chain triglycerides; LPS, lipopolysaccharide; MCT, medium-chain triglycerides; MIP-2, macrophage inflammatory protein; PGE2, prostaglandin E2; TNF-α, tumor necrosis factor alpha; TxB2, thromboxane B2.

Next, we assessed the influence of the different lipid emulsions on the generation of several pro-inflammatory cytokines after the onset of ARDS. The concentration of TNF-α in BAL was determined at 72 pg/ml ± 11 pg/ml under baseline conditions in the NaCl group; comparable levels were detected in animals receiving the different lipid emulsions (Figure 3A). For all treatment groups (*, NaCl,**, LCT; ***, LCT/MCT; § LCT/MCT/FO; *P* <0.05), we could measure a significant increase of TNF-α concentrations 4 h after ARDS induction (4,716 ± 499 pg/ml; NaCl), followed by a steady decrease at 24 h (303 ± 50 pg/ml; NaCl) and 48 h (218 ± 25 pg/ml; NaCl) after LPS-stimulation. After 24 h of injury, mice receiving LCT/MCT/FO showed significantly reduced TNF-α levels as compared to NaCl and LCT ($, *P* <0.05). Furthermore, at 48 h after ARDS induction, the highest TNF-α concentrations at that time point were found in the NaCl group (%, *P* <0.05).

Similar kinetics was determined for MIP-2 levels in BAL (Figure 3B). Starting with comparable basal concentrations in all groups (64 ± 10 pg/ml; NaCl), a peak was reached 4 h after LPS application (3,338 ± 501 pg/ml; NaCl), followed by a steady decline to baseline concentrations after 24 h (161 ± 11 pg/ml; NaCl) and 48 h (117 ± 22 pg/ml; NaCl) (*, *P* <0.05). Interestingly, under basal conditions, LCT (**, *P* <0.05) and LCT/MCT (***, *P* <0.05) displayed significantly elevated MIP-2 levels compared to NaCl. Highest MIP-2 values were measured after 4 h in all groups (§, *P* <0.05). In line with previous results, animals infused with LCT/MCT/FO displayed the lowest MIP-2 levels 24 h after induction of ARDS compared to the other groups ($, *P* <0.05). After 48 h, highest MIP-2 concentrations were detectable for NaCl in comparison to LCT and LCT/MCT/FO (%, *P* <0.05).

Under baseline conditions, thromboxane (Tx)B_2_ concentration in BAL fluid was 31 ± 5 pg/ml without significant differences irrespective of the infused lipid emulsions (Figure 3C). Similar results could be observed 4 h (35 ± 3 pg/ml; NaCl) after LPS stimulation. A significant increase of TxB2 in the NaCl group was only observed after 24 h compared to baseline (*, *P* <0.05). A total of 24 h and 48 h after induction of ARDS, TxB2 was increasing significantly in the LCT (**, *P* <0.05) and LCT/MCT (***. *P* <0.05) groups compared to baseline conditions. After 24 h we could detect significantly reduced TxB2 levels in the LCT/MCT/FO group as compared to the LCT and LCT/MCT groups ($, *P* <0.05). After 48 h, the highest TxB2 concentrations were measured in animals receiving LCT/MCT compared to NaCl and LCT/MCT/FO (%, *P* <0.05).

Prostaglandin (PG)E2 displayed comparable concentrations 0 h (33 ± 6 pg/ml; NaCl) and 4 h (15 ± 1 pg/ml; NaCl) after induction of ARDS with comparable levels in all group (Figure 3D). PGE2 values after 24 h (371 ± 155 pg/ml; NaCl) and 48 h (213 ± 39 pg/ml; NaCl) were significantly elevated in all treatment groups compared to 0 h and 4 h (*, *P* <0.05). Mice treated with LCT/MCT/FO showed a significantly reduced PGE2 concentration 48 h after induction of ARDS as compared to NaCl and LCT ($, *P* <0.05).

### Analysis of fatty acids in plasma

In order to study the effect of the different lipid emulsions under conditions of ARDS on the composition of plasma free fatty acids, plasma concentrations of eicosapentaenoic acid (EPA, 20:5), docosahexaenoic acid (DHA, 22:6), linoleic acid (LOA, 18:2), arachidonic acid (AA, 20:4) and oleic acid (OA, 18:1) were determined by gas chromatography.

**Figure 4:**
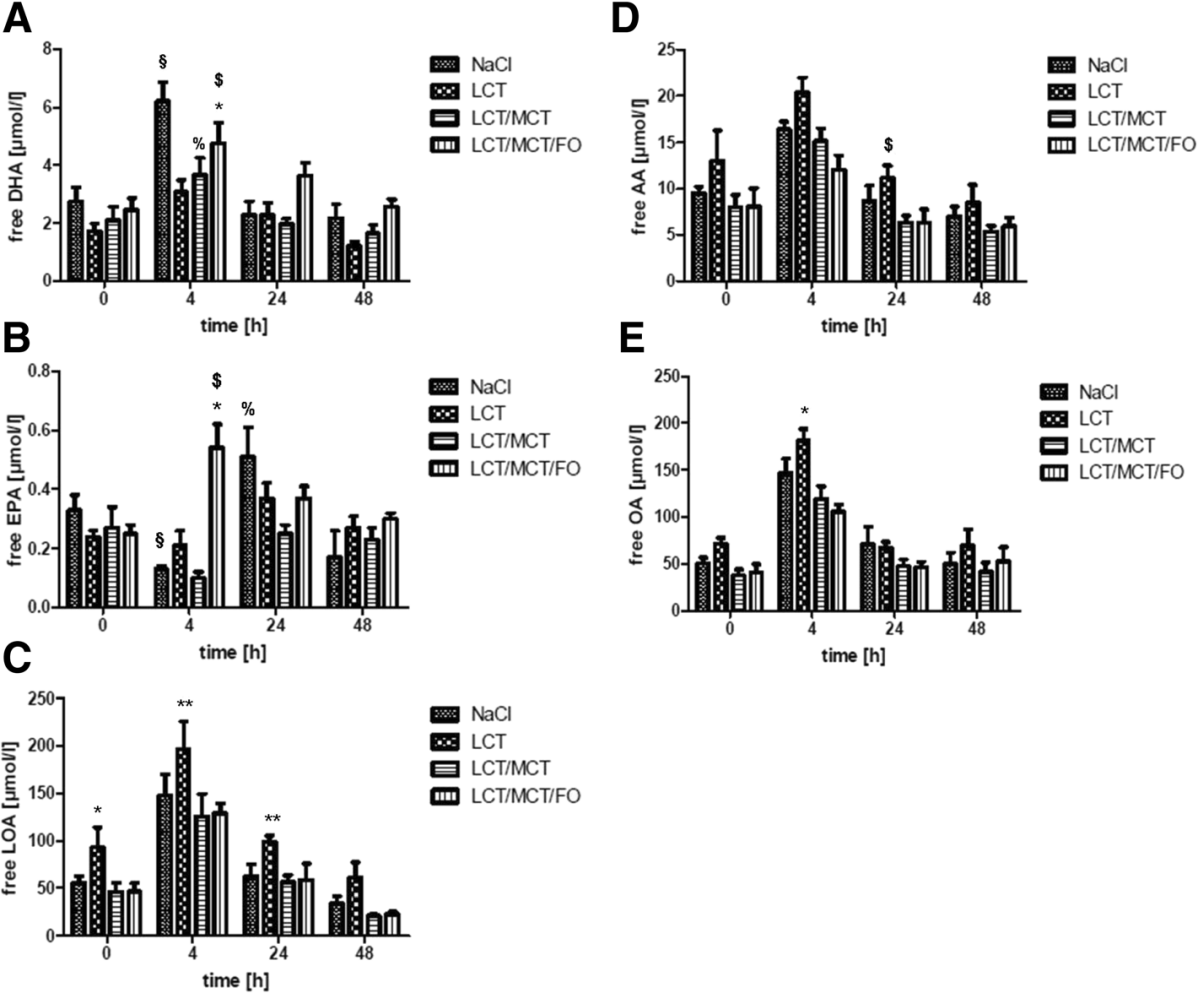
**Free fatty acids in plasma after infusion of lipid emulsions in experimental ARDS. A**. Mice receiving LCT/MCT/FO displayed significantly elevated EPA levels 4 h after LPS stimulation as compared to NaCl, LCT and LCT/MCT (*, *P* <0.05) and to EPA levels at other time points ($, *P* <0.05). EPA concentrations in the NaCl group were the lowest 4 h after induction of ARDS compared 0 h and 24 h (§, *P* <0.05) and after 24 h the highest compared to all time points (%, *P* <0.05). **B**. A total of 4 h after induction of ARDS, the highest DHA levels were measured in the LCT/MCT/FO group (*, *P* <0.05) compared to all time points and to animals receiving LCT and LCT/MCT ($, *P* <0.05). Highest DHA levels in the NaCl group (§, *P* <0.05) and LCT/MCT group (%, *P* <0.05) could be detected after 4 h of LPS stimulation. **C**. Mice infused with LCT had significantly higher LOA levels at baseline compared to NaCl and LCT/MCT/FO (*, *P* <0.05) After 4 h and 24 h, LOA concentrations in mice receiving LCT were the highest compared to the other treatment groups (**, *P* <0.05). **D**. A total of 24 h after LPS challenge, AA concentrations in animals infused with LCT were significantly higher than in the LCT/MCT/FO group ($, *P* <0.05). **E**. After 4 h, LCT-infused mice had significantly elevated OA concentrations as compared to NaCl, LCT/MCT and LCT/MCT/FO (*, *P* <0.05). Data are given as mean ± SEM (n = 6 to 8 independent experiments each). AA, arachidonic acid; ARDS, acute respiratory distress syndrome; DHA, docosahexaenoic acid; EPA, eicosapentaenoic acid; FO, fish oil; LCT, pure long-chain triglycerides; LOA, linoleic acid; LPS, lipopolysaccharide; MCT, medium-chain triglycerides; OA, oleic acid.

Mice receiving LCT/MCT/FO displayed significantly elevated EPA levels 4 h after LPS stimulation as compared to NaCl, LCT and LCT/MCT (*, *P* <0.05) and to EPA levels at other time points ($, *P* <0.05) (Figure 4A). Interestingly, EPA concentrations in the NaCl group were the lowest 4 h after induction of ARDS compared to 0 h and 24 h (§, *P* <0.05) and the highest after 24 h compared to all time points (%, *P* <0.05). Similar results could be observed for DHA. Four hours after induction of ARDS, we could measure the highest DHA levels within the LCT/MCT/FO group (*, *P* <0.05) compared to all time points and to animals receiving LCT and LCT/MCT ($, *P* <0.05) (Figure 4B). Furthermore, the highest DHA levels in the NaCl group (§, *P* <0.05) and LCT/MCT group (%, *P* <0.05) could be detected after 4 h of LPS stimulation. Concerning the n-6 fatty acids LOA and AA, similar kinetics could be detected as concentrations peaked for all treatment groups 4 h after LPS application. Mice infused with LCT had significantly higher LOA levels at baseline compared to NaCl and LCT/MCT/FO (*, *P* <0.05) (Figure 4C). After 4 h and 24 h, LOA concentrations in mice receiving LCT were the highest compared to the other treatment groups (**, *P* <0.05). A total of 24 h after LPS challenge, AA concentrations in animals infused with LCT were significantly higher than in the LCT/MCT/FO group ($, *P* <0.05) (Figure 4D). LOA and AA plasma levels in mice receiving LCT/MCT and LCT/MCT/FO were comparable in all groups. Furthermore, the n-9 fatty acid OA displayed highest levels 4 h after LPS application in all groups (Figure 4E). After 4 h, LCT-infused mice had significantly elevated OA concentrations as compared to NaCl, LCT/MCT and LCT/MCT/FO (*, *P* <0.05).

## Discussion

In the present study we investigated the impact of three different commercially available lipid emulsions in a murine model of acute respiratory distress syndrome. We were able to demonstrate that mice treated with a novel lipid emulsion containing LCT/MCT/FO displayed reduced pulmonary leukocyte invasion, protein leakage and cytokine generation compared to animals receiving LCT or LCT/MCT. Furthermore, it became evident that the combination of the LCT and MCT showed no benefit compared to LCT in LPS-induced lung injury despite our observation of reduced levels of the n-6 fatty acids linoleic acid and arachidonic acid in the plasma of mice receiving LCT/MCT.

A striking finding of our study is the clear immunomodulatory effect of FO-containing lipid emulsions on the development and progression of a LPS-induced acute lung injury. Several biological mechanisms may have contributed to this beneficial effect of FO. Increased provision of n-3 fatty acids (Fas), EPA and DHA, as mirrored in the plasma, may result in augmented incorporation of these FAs into the cell membrane phospholipids and thereby partially replace AA [7,18]. Of note, the highest DHA concentrations were found in the NaCl group at 4 h after ARDS-induction, resembling a feature found in wild type and fat-1 mice [17]. Instead, EPA concentrations were increased in the LCT/MCT/FO group at this time point. It is open to speculation whether infusion of lipids interfered with the generation of free DHA in the course of injury in the groups receiving lipid emulsions and how provision of LCT/MCT/FO increased availability of releasable EPA. On the other hand, AA-derived eicosanoids, such as TxB_2_ and PGE_2_, are generated to a lesser extent in mice receiving n-3 rich FO compared to LCT and LCT/MCT. Besides alterations in eicosanoid synthesis, n-3-induced changes in membrane composition interfered with phospholipid-derived second messengers involved in cell signal transduction, such as formation of inositolphosphates [19], phosphatidylinositol 3-kinase-dependent signaling [20] or activation of protein kinase C [21]. Camandola and colleagues were able to demonstrate that n-6 FAs, unlike EPA, stimulate nuclear translocation and subsequent activation of the transcription factor NF-κB, a key mediator of inflammatory processes [22]. The finding of reduced levels of TNF-α and MIP-2, the murine equivalent of IL-8, in the BAL fluid of LCT/MCT/FO-treated mice in our study can be thus explained by the above-mentioned pathomechanisms.

In our study, intrapulmonary invasion of leukocytes as a characteristic feature of ARDS was reduced in mice receiving FO-containing lipid emulsions, whereas LCT or LCT/MCT increased pulmonary recruitment of neutrophils and lung injury. This finding is strengthened by the observation of reduced MPO activity in lung tissue of FO-treated mice after induction of ARDS. The differential effect of n-3 FA transmigration of neutrophils through the endothelial-epithelial barrier is complex and only partly understood. The n-3 lipids may regulate this multistep process via a reduced presentation of endothelial adhesion molecules [23,24] and the attenuation of the rolling of monocytes [25,26].

Besides the aforementioned properties of n-3 FA in the context of inflammation and ARDS, the recent discovery of novel EPA- and DHA-derived lipid mediators called resolvins represents a milestone in this field of research as these molecules have been found to play a major role in repair and resolution of inflammation [27-29]. A potential impact of resolvins on ARDS remains thus far speculative as the detection of resolvins in BAL fluid in the model of LPS-induced lung injury is difficult to achieve [17].

Direct comparison between the LCT and LCT/MCT results of our study may contribute to the on-going controversial debate on the immunological role of MCT in inflammation. The rationale for implementation of MCT-based lipid emulsions in nutrition regimes are their biochemical properties allowing easy accessibility for metabolic degradation and rapid transport into cells without transport systems [7,10,30]. With respect to the influence of MCT on the immune system, published data have failed to produce a uniform picture. In the experimental setting, LCT/MCT and MCT increase monocyte adhesion and degranulation [31,32], whereas another study reported decreased neutrophil capacity to kill fungal pathogens [33]. The few existing clinical studies demonstrate on the one hand a beneficial effect of LCT/MCT on the development of abscesses after laparotomy [34] but on the other hand aggravated tissue inflammation in patients with ARDS [35]. The data of our present study suggest a similar effect of LCT compared to LCT/MCT in many respects. Our results emphasize the hypothesis that the influence of lipid emulsions on immunological or biological functions are at least in part determined by their n-6 to n-3 ratio which is identical between LCT (7:1) and LCT/MCT (7:1).

Although this is the first report of continuous infusion of LCT/MCT/FO in murine ARDS, our observations are consistent with previous experimental and clinical studies. Recently, our group demonstrated that fat-1 mice, which possess the ability to endogenously convert n-6 to n-3 FA and thus exhibit high levels of EPA/DHA, display reduced features of ARDS and inflammation as compared to wild-type mice [17]. Heller and colleagues conducted a database analysis in 661 intensive care patients with a major fraction suffering from abdominal sepsis [5]. The authors could demonstrate that supplementation of FO into a parenteral nutrition regime exhibits a dose-dependent reduction in length of stay on an ICU and antibiotic demand. Furthermore, in patients receiving FO (0.15 to 0.25 g/kg/d) mortality was decreased significantly [5]. Although the study was neither controlled nor randomized, these findings demonstrated a clinical benefit from the incorporation of FO in parenteral nutrition regimes of critically ill patients. Further evidence for the positive effects of FO was recently provided by a randomized controlled study applying FO-containing lipid emulsions to 40 patients with acute severe pancreatitis [36]. Patients in the n-3 group displayed improved CRP levels and oxygenation indices compared to patients treated with standard lipid emulsions. One of the most recent clinical trials evaluated the effect of a FO-containing lipid emulsion on the immunological and clinical outcome of septic patients [37]. Inclusion of FO in parenteral nutrition provided to septic patients led to altered inflammatory cytokine concentrations and improved gas exchange, whereas the length of stay on the ICU and mortality did not differ among the different groups [37].

It is intriguing that thus far no clinical data have been published on the impact of parenterally applied FO-containing lipid emulsions on the clinical outcome of ARDS. The lack of valid studies in the field of parenteral nutrition in ARDS reinforces the on-going debate on an optimal lipid supply to these patients.

Unlike the parenteral use of n-3 fatty acids in acute lung injury, the enteral application of FO-containing lipids is more fully evaluated thus far. The first multi-centered, controlled and randomized study by Gadek and colleagues investigated the effect of enteral FO-containing immunonutrition on the clinical outcome of 146 patients with ARDS [38]. Patients receiving FO in combination with some other active components showed a significantly improved oxygenation and reduced recruitment of neutrophils in the BAL. Also, non-pulmonary parameters as length of stay on ICU or incidence of new organ failures were improved in the FO-treatment group. In line with these findings, further clinical trials with patients suffering from ARDS [18] and sepsis [11,39] could demonstrate the beneficial effect of n-3 fatty acids partly supplemented with gamma-linolenic acid on oxygenation, duration of mechanical ventilation and organ function. However, the published trials evaluated only patients with continuous full enteral feeding and used a control diet high in n-6 lipids. In contrast, the results of the OMEGA trial conducted by the ARDSnet, posed a challenge [12]. This large phase III trial testing the effect of bolus application of n-3 fatty acids and immuno-supplements in ARDS was stopped due to lack of efficiency and a higher rate of complications in the group of ARDS patients receiving n-3 fatty acids. The supplementation of higher amounts of proteins in the control group and bolus application of n-3 fatty acids leading to possible changes in bioavailability were a matter of debate [40]. In line with the findings of the OMEGA trial, Stapleton and colleagues also could not find a beneficial effect of enterally applied FO on pulmonary and systemic inflammation on patients with ARDS [41].

In conclusion, the use of n-3 fatty acids, especially in patients with acute lung injury, still bears uncertainties due to a lack of studies. Future investigations are needed, focusing on the mode of application (enteral vs. parenteral/bolus vs. continuous), the identification of an optimal dose of n-3 fatty acids, and the best time-point for starting n-3-based nutrition regimes.

In spite of the limitation of our study as to the use of the LPS-induced model of ARDS, which is evidently different from clinical and experimental ARDS induced by bacterial infections, our data clearly demonstrate the beneficial effects of n-3 fatty acids applied parenterally in murine ARDS.

## Conclusion

Fish-oil containing lipid emulsions exert anti-inflammatory and pro-resolving effects in the murine model of LPS-induced ARDS. Thus, prophylactic integration of n-3 fatty acids into a nutritional regime of patients expecting a trauma/operation might be beneficial in reducing pulmonary inflammatory complications.

## Key messages

• Lipid emulsions commercially available for parenteral application exert different immunological effects in a murine model of LPS-induced ARDS.

• Lipid-emulsions rich in n-6 fatty acids appear to increase alveolar recruitment of leukocytes and accumulation of neutrophils in lung tissue after LPS-challenge.

• Fish-oil containing lipid emulsions display anti-inflammatory and pro-resolving properties after LPS stimulation.

• No significant difference was observed between mice infused with LCT or with the combination of LCT and MCT.

## Abbreviations

AA: Arachidonic acid; ANOVA: Analysis of variance; ARDS: Acute respiratory distress syndrome; BAL: Bronchoalveolar lavage; DHA: Docosahexaenoic acid; ELISA: Enzyme-linked immunosorbent assay; EPA: Eicosapentaenoic acid; FO: Fish oil; ICU: Intensive care unit; LCT: Long chain triglycerides; LOA: Linoleic acid; LPS: Lipopolysaccharide, endotoxin; MCT: Medium chain trigycerides; MIP: Macrophage inflammatory protein; M/M: Monocytes/macrophages; MPO: Myeloperoxidase; NF: Nuclear factor; OA: Oleic acid; PG: Prostaglandin; PMN: Neutrophils; PUFA: Polyunsaturated fatty acids; SEM: Standard error of the mean; TNF: Tumor-necrosis factor; Tx: Thromboxane.

## Competing interests

KM has received fees for product-neutral presentations from Abbot, Baxter, BBraun, Fresenius Kabi and Nestlé. The other authors declare that they have no competing interests.

## Authors’ contributions

MH made substantial contributions to the conception, analysis and interpretation of the study and its results. He wrote major parts of the manuscript. JO performed substantial parts of the experiments, participated in analysis and interpretation of the data, and wrote parts of the manuscript. MO performed and analyzed computed tomography experiments, and contributed to the writing of the manuscript. MBS, AH, RM, IV, SH, BR, MW and WS provided intellectual input into the conception and design of the study and the writing of the manuscript. KM supervised the study design and the experiments performed. Furthermore, he was strongly involved in data interpretation and the writing of the manuscript. All authors have given final approval of the manuscript version to be published.

## Authors’ information

MH, IV, SH, RM, WS and KM are members of the German Center for Lung Research (DZL, Deutsches Zentrum für Lungenforschung), and are supported by DZL, Deutsche Forschungsgemeinschaft (SFB Transregio 84), BMBF (KliFo Pneumonie).
